# The effect of marination on lactic acid bacteria communities in raw broiler fillet strips

**DOI:** 10.3389/fmicb.2012.00376

**Published:** 2012-10-18

**Authors:** T. T. Nieminen, H. Välitalo, E. Säde, A. Paloranta, K. Koskinen, J. Björkroth

**Affiliations:** ^1^Department of Food Hygiene and Environmental Health, Faculty of Veterinary Medicine, University of HelsinkiHelsinki, Finland; ^2^DNA sequencing and Genomics Laboratory, Institute of Biotechnology, University of HelsinkiHelsinki, Finland

**Keywords:** *Leuconostoc*, broiler, poultry, lactic acid bacteria, spoilage, marination

## Abstract

Marination with marinade containing salt, sugar, and acetic acid is commonly used in Finland to enhance the value of raw broiler meat. In this study, we investigated the effect of marination, marinade components and storage time on composition of bacterial communities in modified atmosphere-packaged (MAP) broiler fillet strips. The communities were characterized using two culture-independent methods: 16S rRNA gene fragment sequencing and terminal restriction fragment length polymorphism. In unmarinated broiler fillet strips, *Lactococcus* spp. and *Carnobacterium* spp. predominated at the early storage phase but were partially replaced by *Lactobacillus* spp. and *Leuconostoc *spp. when the chilled storage time was extended. In the marinated fillet strips, *Lactobacillus* spp. and *Leuconostoc* spp. predominated independent from the storage time. By mixing the different marinade components with broiler meat, we showed that marination changed the community composition and favored *Leuconostoc* spp. and *Lactobacillus *spp. by the combined effect of carbohydrates and acetic acid in marinade. Marination increased the maximum level of lactic acid bacteria in broiler meat and enhanced CO_2_ production and acidification of meat during the chilled storage. Accumulation of CO_2_ in package head-space due to the enhanced growth of *Leuconostoc* spp. in marinated meat may lead to bulging of packages, which is a spoilage defect frequently associated with marinated and MAP raw broiler preparations in Finland.

## INTRODUCTION

Marination is a commonly used process in the poultry industry because it tenderizes meat, improves sensory quality of meat, and improves raw yield at the processing plant (Smith and Acton, 2000; Björkroth, 2005). In Finland, most raw poultry sold at the retail level is marinated by brine injection followed by blending with mixture of oil, water, spices, vinegar, and glucose or sucrose and packaged under modified atmosphere (MA) containing N_2_ and CO_2_.

Modified atmosphere-packaged (MAP), marinated broiler preparations have been shown to spoil due to extensive bulging of packages caused by *Leuconostoc gasicomitatum* (Björkroth et al., 2000). A study by Susiluoto et al. (2003) confirmed that *L. gasicomitatum* and *Leuconostoc gelidum* predominate in Finnish marinated broiler fillet strips on the use-by date. *L. gasicomitatum* and *L. gelidum* have also been associated with spoilage of other types of muscle food supplemented with glucose or sucrose (Lyhs et al., 2004; Vihavainen and Björkroth, 2007). Due to the relevance of *Leuconostoc* spp. as spoilage organisms, identifying the factors that promote the growth of *Leuconostoc *spp. in marinated poultry preparations is of interest and could provide means to spoilage control. In a recent study, we showed that marination of broiler fillet strips increased the proportions on *L. gasicomitatum* and *L. gelidum* in late shelf-life phase bacterial communities in comparison to unmarinated broiler fillet strips that were predominated by *Carnobacterium* spp. (Nieminen et al., 2012). However, the effect of meat storage time on the relative abundance of *Leuconostoc* spp. was not elucidated and the marinade components that favored the growth of *Leuconostoc* spp. in the marinated broiler preparation were not identified.

In this study, our aim was to find factors explaining the predominance of *Leuconostoc* spp. in late shelf-life marinated broiler meat. To achieve this, we compared bacterial succession in marinated and unmarinated broiler fillet strips and identified the marinade components that enriched *Leuconostoc* spp. in the bacterial communities.

## MATERIALS AND METHODS

### BROILER MEAT

This study comprised of two test series, referred as Experiment 1 and Experiment 2, which utilized different broiler meat samples and partly different experimental set-up.

For Experiment 1, we purchased 10 consumer packages (300 or 650 g) of marinated and 10 consumer packages of unmarinated MAP broiler fillet strips from the local supermarkets within a period of 1 month. All packages originated from the same factory but represented different manufacturing lots. The packages were purchased at least 6 days before the end of the manufacturer-defined shelf-life, a period which was 9 or 10 days in total. The unmarinated strips contained only meat. Marinated strips contained 0.068% acetic acid (Wt/Wt), 0.68% honey, 0.66% glucose, 0.82% maltodextrin, 0.89% NaCl, 0.2% phosphate, 3.0% rape seed oil, 0.26% spices (sweet pepper, curry, black pepper, garlic, and turmeric), 0.34% thickeners (guar gum and xanthan gum), and 0.06% yeast extract.

The packages were brought to laboratory immediately after purchase, opened and re-packaged under MA (40% CO_2_ and 60% N_2_) in a 4 L bag with oxygen barrier (Amilen Ox 90, Finnvacum, Helsinki, Finland). The packages were stored in the dark at +6 ± 0.5°C. At the time of sampling, the packages were opened aseptically and 10 or 25 g of strips were collected, depending on the analyses. The remaining strips were re-packaged under MA of original composition and stored at +6°C until the next sampling. This non-destructive sampling approach enabled us to monitor the same bacterial communities throughout the storage period instead of measuring communities separated from each other, which would have been the result of dividing the strips to individually packaged aliquots measured at different time points.

Experiment 2 was designed to investigate the effect of the marinade components on the composition of the bacterial communities. MAP marinated broiler fillet strips and MAP brined fillet strips were obtained directly from the factory. The brined strips contained 15% (Wt/Wt) of brine and 0.6% (Wt/Wt) of NaCl. The composition of marinated strips was the same as in Experiment 1. The strips for Experiment 2 arrived to the laboratory 1 day after packaging in the factory. At the laboratory, the brined strips were unpackaged, pooled, and divided to eight portions. Each of the portions was mixed with a different components of marinade (**Table 1**) and divided to four parallel portions (150 g each) which were packaged individually under MAP (40% CO_2_ and 60% N_2_) in a 4 L bag with an oxygen barrier. The packages were stored in dark at +6 ± 0.5°C and analyzed separately. The factory-packaged marinated (MAF) and brined (BRF) fillet strips were investigated together with the laboratory-packaged strips.

**Table 1:** The marinade components mixed with brined fillet strips.

Treatment name	Components added per 100 g of brined strips
BRL/BRF	No additions (BRL packaged in the laboratory, BRF packaged in the production plant)
H2O	9.76 g water, 3.53 g rape seed oil
HCL	1.33 ml of 1 M HCl*
SPI	Spice mixture containing 0.31 g spices (sweet pepper, curry, black pepper, garlic, and turmeric), 0.4 g thickeners (xanthan gum and guar gum), and 0.070 g yeast extract*
ACE	0.79 ml of 10% acetic acid*
CAR	Carbohydrates: 0.95 g of glucose, 0.79 g of honey, and 0.98 g of maltodextrin (dextrose equivalent 13–17, Sigma-Alrich, St Louis, MO, USA)*
A&C	Acetic acid and the carbohydrates*
MAL/MAF	All the components of the marinade (MAL packaged and marinated in the laboratory, MAF packaged and marinated in the factory)

* The components were added to the strips together with 9.76 g of water and 3.53 g of rape seed oil.

### COLONY COUNTS AND pH

The colony counts were measured from broiler meat as described before (Nieminen et al., 2012) on Man–Rogosa–Sharpe agar (MRS, Oxoid, Basingstoke, UK, pH 6.2) for LAB, Violet Red Bile Glucose agar (VRBG, Lab M, Bury, UK) for enterobacteria and Streptomycin sulfate thallium acetate actidione agar (STAA Oxoid, Basingstoke, UK) for *Brochothrix thermosphacta*. The incubation temperature was 25°C for all bacteria. Meat pH was measured from meat homogenate by an inoLab pH 720 (WTW GmbH, Weilheim, Germany) instrument.

### TERMINAL RESTRICTION FRAGMENT LENGTH POLYMORPHISM ANALYSIS

Bacterial communities in meat were characterized culture-independently by terminal restriction fragment length polymorphism (T-RFLP). For Experiment 1, each broiler fillet strip package was sampled twice during the chilled storage period in the laboratory. The first samples were collected after 46–141 h of storage in the laboratory and represented communities at the early storage phase. The second samples were collected after 260–385 h of storage and represented samples at the late storage phase. The sampling times varied from package to package because our aim was to select the sampling points based on the LAB colony counts and growth curves rather than storage times. This approach was chosen because the colony counts in different packages were not equal when the packages were brought to the laboratory.

For the Experiment 2, the bacterial communities in fillet strips mixed with different marinade components were sampled for the T-RFLP analysis once after 13 or 14 days of storage at +6°C.

To characterize the bacterial communities, bacterial DNA was extracted from the fillet strips as describe before (Nieminen et al., 2012). Two parallel extracts were collected from each package and analyzed separately. The DNA extracts were used as a template for PCR amplification of approximately 940-bp 16S rRNA gene fragment with forward primer Bact-8F (5′-AGA GTT TGA TCC TGG CTC AG) and reverse primer 926r (5′-CCG TCA ATT CMT TTG AGT TT). PCR, restriction digestion with enzymes *Hpy*8I, *Hin*P1I, and *Nla*IV and capillary electrophoresis was performed as described earlier (Nieminen et al., 2011).

The T-RFLP data was imported to Peak Scanner software (version 1.0, Applied Biosystems, Foster City, CA, USA). The peaks were identified using zero as the peak-height cut-off value and 50–1000 bp as the size range. The resulting peak table was imported to T-REX software (Culman et al., 2009) which was used to filter noise (standard deviation three, use peak height), align peaks (clustering threshold 0.5, at most one peak per profile), average parallel DNA extracts, and create a data matrix [meat packages vs. aligned terminal restriction fragments (T-RFs)]. The values in the data matrix represented relative abundances of the T-RFs and were calculated by dividing each peak height by the sum of peak heights measured from the same sample.

For the ordination analyses of the communities, the T-RFLP data matrices obtained with the three restriction enzymes were combined to a single data matrix and a distance matrix based on Jaccard index was calculated. The distance matrix was used for non-metric multidimensional scaling (NMDS) that was performed in R software environment (v. 2.12.1) using the functions in the library vegan (Oksanen et al., 2011). NMDS was performed using the functions metaMDSiter and postMDS. For each treatment (the four combinations of storage phase and marination) a centroid in the ordination space was calculated with the function Envfit. Function Ordiellipse was used to draw ellipses that illustrated standard deviations of the community structures within each treatment.

To assign the T-RFs to bacterial groups, we compared the T-RF lengths to those measured from known meat-associated strains (Nieminen et al., 2011) and the strains *Vagococcus fessus* DSM 15697^T^, *Vagococcus carniphilus* DSM 17031^T^, *Vagococcus fluvialis* DSM 5731^T^, *Vibrio litoralis* DSM 17657^T^, and *Vibrio rumoiensis* DSM 19141^T^. To obtain a value for relative abundance of bacterial group in meat package, the relative abundances of the three T-RFs corresponding to same bacterial group were averaged. When several bacterial groups generated a T-RF of equal length, the corresponding T-RF was excluded from the average.

### 16S rRNA GENE PARTIAL SEQUENCING

The phylogenetic structures of bacterial communities in marinated and unmarinated meat (Experiment 1) at early and late storage phases were investigated by partial 16S gene sequencing. The V1–V3 regions of the 16S rRNA gene were amplified in three technical replicates, each using the same DNA template, with the universal bacterial primers pA′ (AGAGTTTGATCMTGGCTCAG; Weisburg et al., 1991) and pD′ (GTATTACCGCGGCTGCTG; Edwards et al., 1989). The DNA templates for PCR reactions were constructed by pooling equal amounts of DNA from the 10 community DNA samples obtained from each of the four treatments (marinated and unmarinated broiler fillet strips at early and late storage phases). Details of the cycling conditions, PCR protocol, and PCR-product processing were described previously (Koskinen et al., 2011). Sequencing was done with the 454 Genome Sequencer FLX using GS FLX Titanium series reagents [Roche/454 Life Science, (Margulies et al., 2005)]. Sequencing and the quality control of the obtained sequences were described in more detail earlier (Nieminen et al., 2012). The quality-controlled sequences were assigned to bacterial taxa with the Mothur program (Schloss et al., 2009) using NCBI taxonomy and a Bayesian method. An 80% identity was required for genus level assignment.

## RESULTS

### THE EFFECT OF MARINATION AND STORAGE TIME ON BACTERIAL COMMUNITIES IN BROILER FILLET STRIPS

The T-RFLP method was used to investigate the effect of marination on the commercial broiler fillet preparations at the early and the late storage phases. LAB colony counts at both phases were measured to characterize the communities and were as follows (log CFU g^–1^ ± standard deviation): unmarinated, early phase 5.7 ± 1; unmarinated, late phase 8.5 ± 0.1; marinated, early phase 5.8 ± 0.7; marinated, late phase 8.8 ± 0.3. The sampling points at the late storage phase in relation to the LAB growth curves can be seen in **Figure 3**: the late samples were collected at the last time point of each curve. The growth curves in **Figure 3** illustrate that the samples at the late storage phase were collected from both marinated and unmarinated broiler fillet strips approximately at the same growth phase of LAB and were comparable in this respect.

**FIGURE 1:**
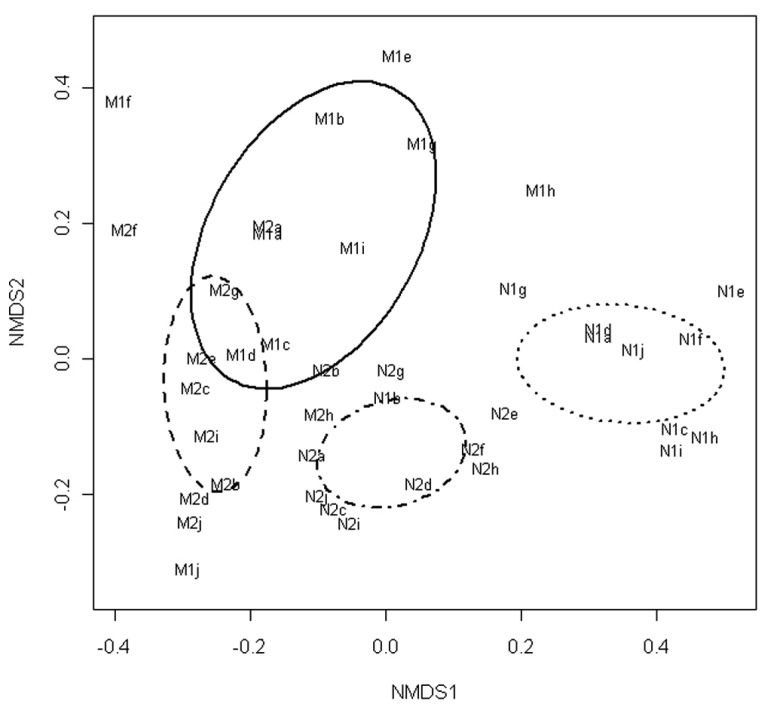
**Non-metric multidimensional scaling of T-RFLP data obtained from bacterial communities in marinated (M) and natural (N) broiler fillet strips at the early (1) and the late (2) phase of chilled storage**. Ellipses represent standard deviations of the community structures.

The NMDS of the T-RFLP data grouped the communities according to the marination treatment and storage phase (**Figure 1**). Fitting of the centroids of the two factors (storage phase and marination) separately to the ordination resulted an *R*^2^ value of 0.22 and *P*-value of 0.001 for the storage phase and *R*^2^ value of 0.37 and *P*-value of 0.001 for the marination treatment indicating that both storage phase and marination affected the community composition in the commercial preparations.

**FIGURE 2:**
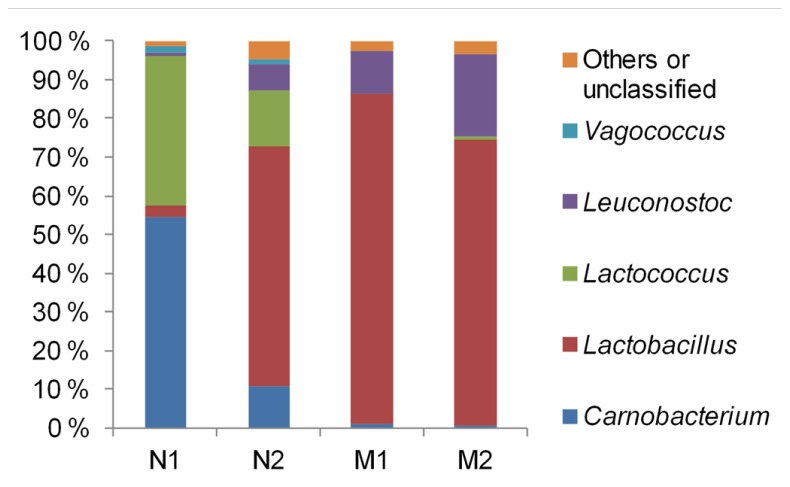
**Phylogenetic structures of bacterial communities in marinated (M) and unmarinated (N) broiler fillet strips at the early (1) and the late (2) storage phases as indicated by 16S rRNA gene fragment sequencing**.

The average community compositions were elucidated by sequencing of the 16S rRNA gene fragments which were amplified from the pooled community DNA samples. We obtained 5920, 8253, 2817, and 5071 sequences from the samples N1, N2, M1, and M2, respectively. Median sequence length of the combined data set was 490 bp. The sequences were assigned almost exclusively to LAB (**Figure 2**). *Vagococcus*, *Lactococcus*, and *Carnobacterium* predominated in unmarinated meat at the early storage phase and were partly replaced by *Leuconostoc* and *Lactobacillus* at the late storage phase. In marinated meat leuconostocs and lactobacilli predominated at both early and late storage phases. Similarly to unmarinated meat, the proportion of *Leuconostoc* spp. in marinated meat appeared to increase at the late storage phase.

### THE EFFECT OF MARINATION ON pH AND LAB COLONY COUNTS

**FIGURE 3:**
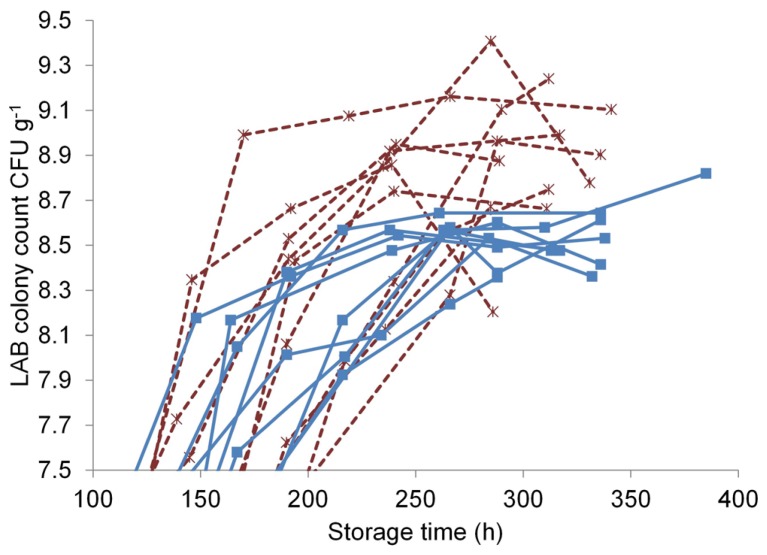
Lactic acid bacterial growth curves in unmarinated (■, solid line) and marinated (*, dashed line) broiler fillet strips stored at +6°C.

Marination increased the maximum LAB colony counts in the broiler fillet strips (**Figure 3**) by an average of 0.41 log CFU g^-1^ (calculated using the highest measured value from each package), corresponding to 2.6-fold difference in colony counts.

**FIGURE 4:**
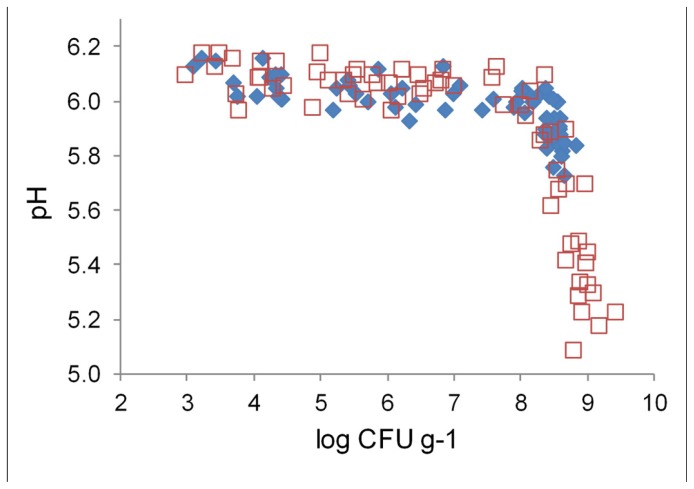
**Correlation between pH and lactic acid bacterial colony counts in marinated (□) and unmarinated (◆) broiler fillet strips**.

Marination did not change initial pH of broiler fillet strips (**Figure 4**). The pH values of marinated and unmarinated meat showed similar correlations with LAB colony counts. When the colony counts remained below 8 log CFU g^-1^, meat pH varied between 5.9 and 6.2, and showed weak negative correlation with the colony counts. When LAB colony counts exceeded 8 log CFU g^-1^, negative correlation between the colony counts and meat pH was much stronger than at lower LAB levels. The pH values at the end of the storage period were up to 0.5 pH units lower in marinated meat compared to unmarinated meat, which had lower final LAB

### THE EFFECT OF MARINADE COMPONENTS ON BACTERIAL COMMUNITIES

The LAB colony counts measured from fillet strips mixed with different components of marinade ranged from 8.2 log CFU g^-1^ to 9.2 log CFU g^-1^ (**Table 2**). The lowest LAB levels were measured from unmarinated brined meat without marinade components (treatments named BRF, BRL, and H2O in **Table 2**). The additions of HCl or acetic acid to the strips did not affect the LAB colony counts but acetic acid decreased the enterobacterial colony counts. The highest LAB levels were measured from the marinated strips (MAF and MAL) and the strips containing the carbohydrate (CAR) or spice components (SPI) of the marinade.

**Table 2:** Colony counts and pH values measured from broiler fillet strips mixed with marinade components (specified in Table 1) and stored at +6°C.

Treatment name	Colony count (log CFU g-1)			pH	
		LAB	*Enterobacteriaceae*	*B. thermosphacta*		
	13 –14 days	13 –14 days	13 –14 days	2 days	13 –14 days
BRF	8.5 ± 0.1	6.3 ± 0.2	5.5 ± 0.2	6.2	6.1 ± 0.0
BRL	8.5 ± 0.1	5.8 ± 0.2	3.7 ± 0.6	6.3	6.0 ± 0.0
H2O	8.4 ± 0.1	5.9 ± 0.1	4.3 ± 1.0	6.3	6.0 ± 0.0
HCL	8.5 ± 0.1	5.8 ± 0.3	4.4 ± 0.3	6.1	5.9 ± 0.0
SPI	8.8 ± 0.1	5.7 ± 0.2	4.2 ± 0.5	6.2	5.9 ± 0.1
ACE	8.4 ± 0.2	4.3 ± 0.1	3.6 ± 0.5	6.1	5.9 ± 0.0
CAR	8.9 ± 0.1	5.1 ± 0.2	4.0 ± 0.5	6.2	5.5 ± 0.0
A&C	8.8 ± 0.1	3.5 ± 0.5	4.0 ± 0.5	6.1	5.5 ± 0.1
MAF	9.1 ± 0.1	3.2 ± 0.2	4.7 ± 0.2	6.0	4.9 ± 0.1
MAL	9.1 ± 0.1	3.6 ± 0.4	3.6 ± 0.4	6.1	5.5 ± 0.4

*The numbers indicate the average ± standard deviation of results obtained from three or four parallel meat packages.Two parallel packages of each sample type were measured after 13 days and the remaining packages after 14 days of storage.*

During the storage, pH dropped by 0.2–0.3 units in preparations without added carbohydrates. When the carbohydrates were added, pH decreased from 0.5 to 1.0 during the storage period.

The head-space CO_2_ concentrations in factory and laboratory-packaged preparations ranged from 52 to 57% in the beginning of the storage period. The CO_2_ concentrations in the laboratory packages remained constant during the storage but increased to 63–67% in the factory packages containing marinated meat and decreased to 37–39% in factory packages containing unmarinated brined fillet strips.

**FIGURE 5:**
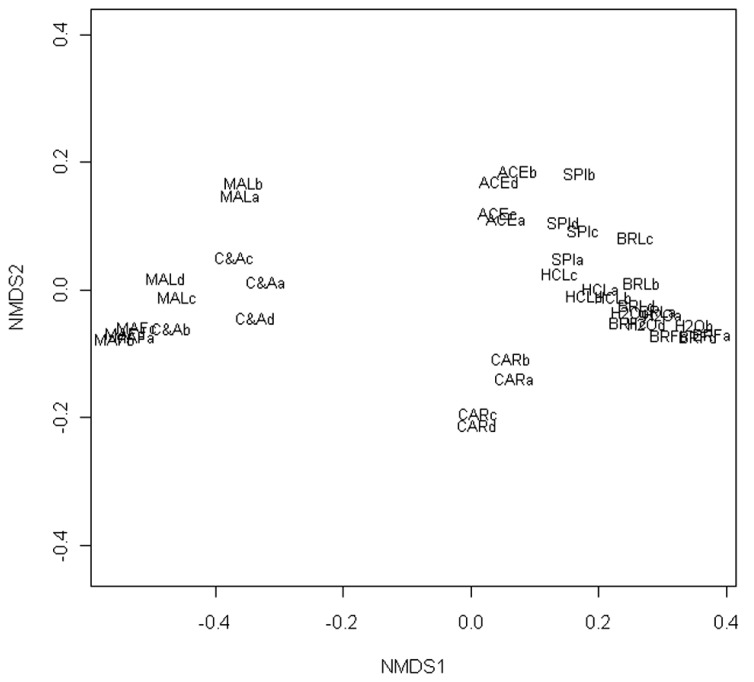
**Non-metric multidimensional scaling plot describing the effect of marinade components on the structures of bacterial communities in marinated and natural broiler fillet strips**. Each data point represents community in a single meat package. Four parallel packages (a–d) were subjected to each of the 10 treatments specified in **Table 1**.

Non-metric multidimensional scaling of T-RFLP results from bacterial communities in meats mixed with different marinade components (**Figure 5**) indicated that addition of spice mixture or lowering of meat pH with HCl did not alter the community structure when compared to the unmarinated brined meat. Addition of sugar or acetic acid changed the community structure but in a different way. When sugar and acetic acid were added together, the community structure was similar to that measured from marinated meat.

**FIGURE 6:**
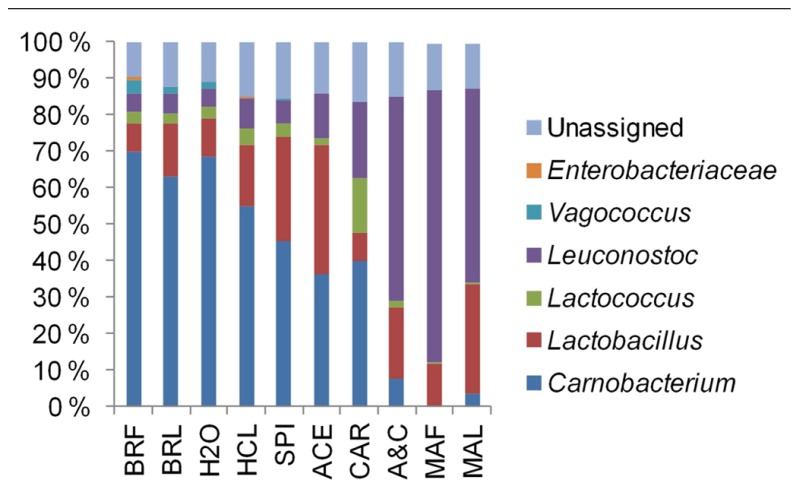
**The phylogenetic structures derived from terminal restriction fragment length polymorphism analysis of bacterial communities in broiler fillet strips mixed with the marinade components specified in Table 1**.

By associating T-RFs with bacterial groups (**Table A1**), we were able to obtain information about phylogenetic structures of bacterial communities in broiler meat. The changes in relative proportions of T-RFs assigned to different LAB (**Figure 6**) indicated that when all components of marinade were added together to fillet strips, proportions of leuconostocs increased and proportions of carnobacteria, vagococci, and lactococci decreased in the communities when compared to unmarinated brined fillet strips. These results were in concordance with the results obtained by 16S rRNA gene fragment sequencing of the communities in a different set of packages (**Figure 2**). As shown in **Figures 5 and 6**, community structure similar to that of marinated broiler fillet strips was obtained by adding carbohydrates and acetic acid together on the strips. Acetic acid alone increased the proportion of T-RFs associated with lactobacilli when compared to unmarinated brined meat (**Figure 6**). Addition of carbohydrates increased the proportions of T-RFs associated with leuconostocs and lactococci but not the proportions of T-RFs associated with lactobacilli.

## DISCUSSION

Bacterial communities in marinated and unmarinated broiler fillet strips were predominated by bacteria belonging to genera *Lactobacillus*, *Lactococcus*, *Leuconostoc*, and *Carnobacterium* that have been frequently associated with MAP raw meats (Doulgeraki et al., 2012). We also identified vagococcal DNA in brined and unbrined, unmarinated broiler fillet strips (**Figures 2 and 6**). Vagococci have been previously detected in salmon (Macé et al., 2012), shrimps (Jaffrés et al., 2009), and ground beef (Shewmaker et al., 2004). The present study and our previous study (Nieminen et al., 2012) show that vagococci are repeatedly a part of the predominating microbiota in late shelf-life Finnish MAP unmarinated broiler fillet strips and that they are inhibited by acetic acid-containing marinade.

As shown before (Nieminen et al., 2012), marination changed the composition of the bacterial communities in broiler fillet strips by favoring leuconostocs and lactobacilli over carnobacteria, vagococci, and lactococci. These differences could not be explained by initial pHs of the preparations, which were not affected by marination. The carbohydrates and acetic acid were identified as the marinade components that were required to change the composition of the communities (**Figure 5**). Lactobacilli were favored by marination because they tolerated acetic acid better than the competing LAB (**Figure 6**). The tolerance of lactobacilli to organic acids could also explain why lactobacilli were able to replace carnobacteria and lactococci when the chilled storage time of unmarinated filled strips was extended (**Figure 2**). Carnobacteria were replaced by leuconostocs and lactobacilli during chilled storage of vacuum-packaged beef (Jones, 2004) and cooked meat products (Chenoll et al., 2007) presumably due to the increasing acidity of meat.

Similarly to the present study, lactococci predominating at the exponential storage phase were partly but not completely replaced by leuconostocs when the communities reached the stationary storage phase in pork (Rahkila et al., 2012) suggesting that this succession pattern occurs frequently in MAP raw meat.

Leuconostocs were favored by marination (**Figures 2** and 6). The results imply that leuconostocs were able to utilize the added carbohydrates more efficiently than the competing LAB, including lactobacilli (**Figure 6**). This could explain why leuconostocs have been frequently associated with the spoilage of muscle food supplemented with glucose or sucrose (Björkroth et al., 2000; Susiluoto et al., 2003; Lyhs et al., 2004; Vihavainen and Björkroth, 2007). Our results indicated also that the proportion of leuconostocs increased in both marinated and unmarinated preparations at the late storage phase. Similar results were obtained by Schirmer et al. (2009) who showed that *Leuconostoc* spp. were enriched in the bacterial communities during the chilled storage of vacuum-packaged marinated pork. Thus, compared to some of their competitors, leuconostocs appear to be resistant to the accumulation of organic acids and/or depletion of readily available carbon sources possibly explaining their frequent occurrence in late shelf-life raw meat (Doulgeraki et al., 2012).

Bacterial communities in marinated broiler meat appeared to be more stable than in unmarinated meat during the chilled storage (**Figures 1 and 2**) although the simultaneous decrease in pH was higher in marinated meat. It is possible that acetic acid included in marinade already selected the most acid tolerant psychrotrophic LAB strains present in the initial microbiota and further selection due to accumulation of organic acids as the function of bacterial growth was minor. An alternative explanation is that intragenus succession occurred in marinated meat but was not detected with the culture-independent methods used in this study.

Marination doubled the maximum LAB colony counts in broiler fillet strips (**Figure 3**). The additional LAB growth in marinated meat resulted in lower final pH of meat (**Figure 4**) and higher CO_2_ concentration in package head-space (**Table 2**) in comparison to unmarinated meat. Acidity decreases solubility of CO_2_ and thus enhances accumulation of CO_2_ to package head-space, possibly leading to blown packages, which is a commonly observed defect in late shelf-life MAP marinated broiler fillet strips in Finland (unpublished observations). Interestingly, pH of the fillet strips correlated with LAB colony counts and was independent from the marination treatment (**Figure 4**) which shows that LAB metabolism during the storage decreased pH more than acetic acid included in the marinade.

In conclusion this study indicated that added acetic acid and carbohydrates and extended chilled storage were the factors that enriched *Leuconostoc* spp. in bacterial communities in MAP broiler meat.

## Conflict of Interest Statement

The authors declare that the research was conducted in the absence of any commercial or financial relationships that could be construed as a potential conflict of interest.

## Appendix

**Table A1:**

Size (bp) of the alignedT-RF			Corresponding taxa in the identification library^1^	Assignment	Enzymes used for quantification
*HinP1I*	*Hpy8I*	*NlaIV*			
584	811	236	*Carnobacterium divergens, C. maltaromaticum*	*Carnobacterium*	All three
370	800	196	*Enterobacteriaceae*	*Enterobacteriaceae*	*HinP1I*
605	841	256	*Lactobacillus oligofermentas*	*L. oligofermentas*	*HinP1I, NlaIV*
603	840	254	*Lactobacillus sakei, L. fuchuensis*	*Lactobacillus*	*HinP1I, NlaIV*
581	216	230	*Lactococcus spp.*	*CLactococcus*	All three
204	113	229	*Leuconostoc gelidum, L. mesenteroides*	*Leuconostoc*	*Hpy8I*
204	648	229	*Leuconostoc gasicomitatum, L. gelidum, L. carnosum*	*Leuconostoc*	*HinP1, NlaIV*
242	816	238	*Vagococcus fessus^2^*	*Vagococcus*	All three

1 Nieminen et al. (2011).

2 Vagococcus fessus DSM 15697T; T-RFs: HinP1I 242 bp, Hpy8I 814 bp, NlaIV 238 bp.
